# Exploring the impact of m^6^A modification on immune diseases: mechanisms and therapeutic implication

**DOI:** 10.3389/fimmu.2024.1387582

**Published:** 2024-07-12

**Authors:** Yutong Chen, Min Liu, Miao Lu, Linling Luo, Zhongyu Han, Xide Liu

**Affiliations:** ^1^ The Second Clinical Medical College, Zhejiang Chinese Medical University, Hangzhou, China; ^2^ Department of Traditional Chinese Medicine, Zhejiang Hospital of Integrated Traditional Chinese and Western Medicine, Hangzhou, Zhejiang, China; ^3^ School of Medical and Life Sciences, Chengdu University of Traditional Chinese Medicine, Chengdu, China

**Keywords:** N6-methyladenosine, M6A, autoimmune diseases, immune cells, T cell

## Abstract

N^6^-methyladenosine (m^6^A) is a chemical modification of RNA and has become a widely discussed topic among scientific researchers in recent years. It is distributed in various organisms, including eukaryotes and bacteria. It has been found that m^6^A is composed of writers, erasers and readers and is involved in biological functions such as splicing, transport and translation of RNA. The balance of the human immune microenvironment is important for human health abnormalities. Increasing studies have found that m^6^A affects the development of immune diseases such as inflammatory enteritis and systemic lupus erythematosus (SLE) by participating in the homeostatic regulation of the immune microenvironment *in vivo*. In this manuscript, we introduce the composition, biological function, regulation of m^6^A in the immune microenvironment and its progression in various immune diseases, providing new targets and directions for the treatment of immune diseases in clinical practice.

## Introduction

**Figure 1:**
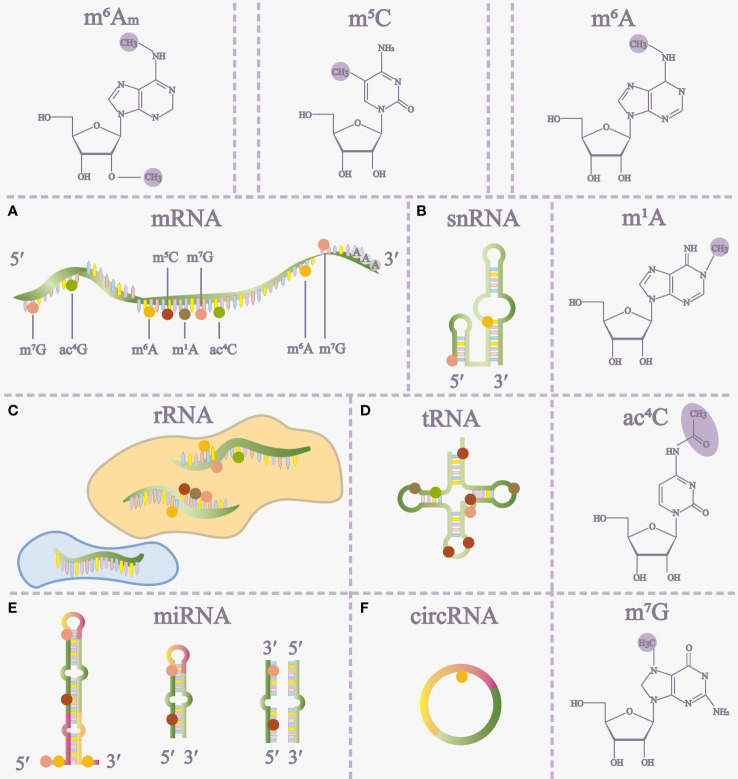
The distribution of RNA modifications on different RNAs **(A-F)** and some RNA modification structures. m^5^C 5-methylcytosine, m^6^A N^6^-methyladenosine, m^1^A N^1^-methyladenosine, ac^4^C N^4^-acetylcytidine, m^7^G 7-methylguanosine, m^6^Am, N^6^, 2’-O-dimethyladenosine, miRNA microRNA.

RNA modifications, as chemical alterations are prevalent across diverse cell types and hold significant relevance in various pathophysiological processes. These modifications include N^6^-methyladenosine (m^6^A), 5-methylcytosine (m^5^C), N^1^-methyladenosine (m^1^A), N^7^-methylguanosine (m^7^G), and N^4^-acetylcytosine (ac^4^C) (1–4) (**Figure 1**). Notably, m^6^A, discovered in 1974, stands out as the most extensively studied RNA modification. Advancements, notably methyl-RNA-immunoprecipitation-sequencing (MeRIP-seq) in 2012, have deepened our understanding of m^6^A’s role in gene expression (5, 6) (**Figure 1**).

m^6^A modifications are pervasive across a wide range of RNA molecules, encompassing both coding and non-coding RNAs. They play pivotal roles in regulating transcription, translation, and RNA decay (7). Growing research links m^6^A methylation to various human diseases. Numerous studies highlight a close connection between m^6^A and the immune microenvironment, emphasizing its role in immune regulation and its impact on autoimmune diseases (ADs).

In this manuscript, we delve into the regulatory role of m^6^A methylation in the immune microenvironment, particularly in immune cells. We focus on the latest research findings regarding the involvement of m^6^A methylation in diverse immune diseases and viral infections. Additionally, we explore the feasibility and challenges of targeting m^6^A as a potential treatment for ADs.

## Modification mechanism of m^6^A

**Figure 2:**
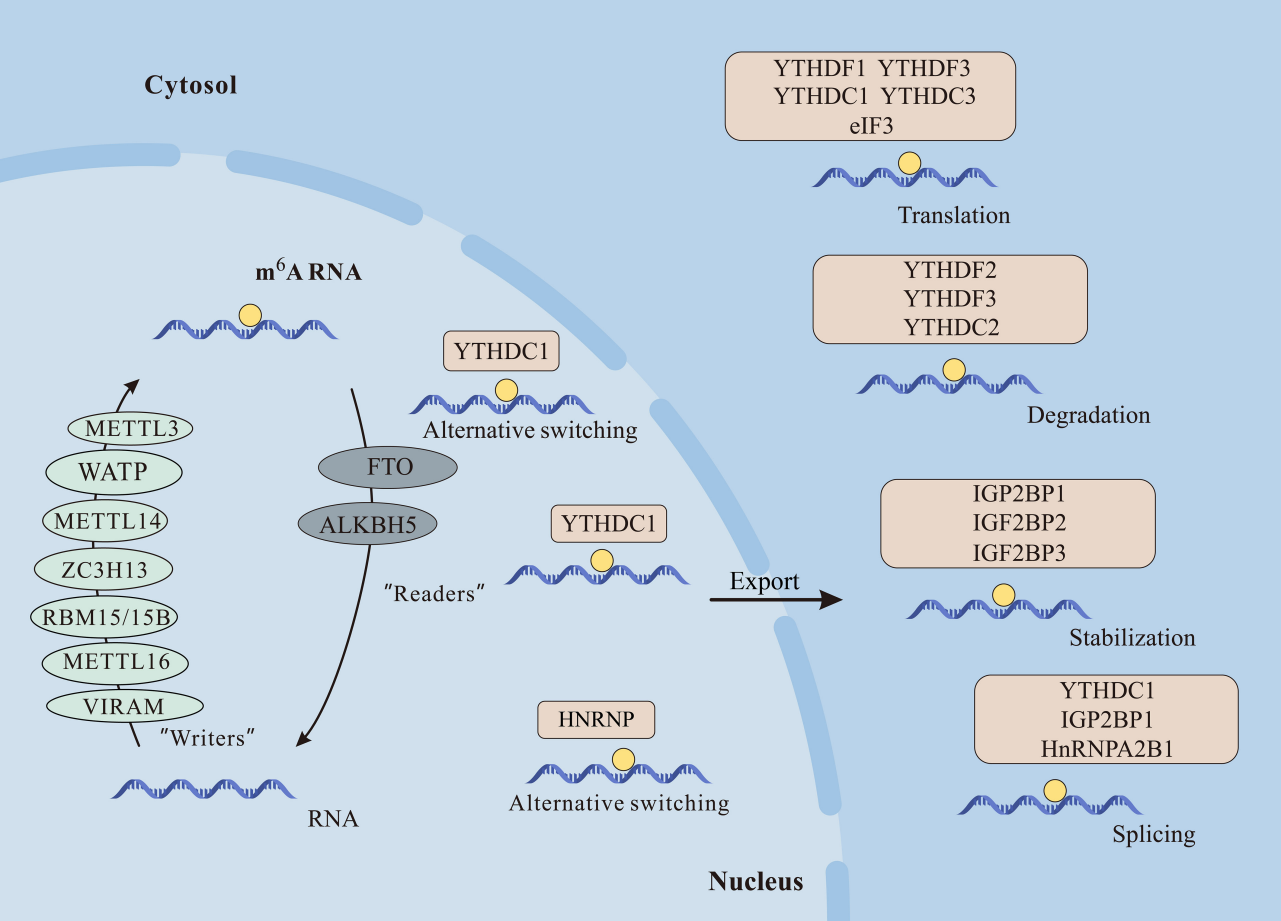
The m^6^A modification plays crucial roles in mRNA function. Writers like METTL3, METTL14, METTL16, WTAP, VIRMA, RBM15/15B, and ZC3H13 catalyze m^6^A methylation. Erasers FTO and ALKBH5 demethylate m^6^A, while readers including YTHDF1/2/3, YTHDC1/2, IGF2BP1/2/3, and HNRNPC/A2B1 recognize m^6^A, influencing mRNA processes like alternative splicing, nuclear export, translation, degradation, and stabilization.

m^6^A illustrates methylation of the adenine base at the nitrogen-6-position and consists of three parts: “writers” (methyltransferase), “erasers” (demethylase), and “readers” (m^6^A-binding protein), in which they are responsible for the addition, removal, and recognition of m^6^A, respectively (8) (**Figure 2**).

### Writers

Writers promote methylation and include methyltransferase-like 3 (METTL3), methyltransferase-like 5 (METTL5), methyltransferase-like 14 (METTL14), Wilms’ tumor 1-associating protein (WTAP), RNA binding motif protein (RBM15), Zinc finger CCCH-type containing 13 (ZC3H13), and protein virilizer homolog (VIRMA) and so on.

Among them, METTL3 was the core component of the m^6^A methyltransferase complex (MTC) first discovered in 1997 and plays a role in promoting translation (9). METTL14 is another component that does not catalyze methyl transfer but can form a heterodimer MTTTL3-METTL14 complex with METTL3 to improve the catalytic activity of m^6^A (10–12). METTL5 is a methyltransferase of 18S ribosomal RNA (rRNA) that forms a heterodimer with transfer RNA (tRNA) methyltransferase subunit 112 (TRMT112), thereby facilitating modification of N6-methylation of m^6^A_1832_ (13). WATP is another core part of MTC, which acts as a regulatory and recruits MTTTL3-METTL14 complex to messenger RNA (mRNA) (14). RBM15 and its analog RBM15B bind the m^6^A methyltransferase complexes to specific mRNAs because of interaction with WATP. They also affect the long non-coding RNA X-inactive specific transcript (XIST) m^6^A methylation (15). ZC3H13 promotes m^6^A modification and it has been shown that reduction of ZC3H13 decreases methylation level of the 3’ untranslated regions (3′UTRs) (16). VIRMA mediates preferential mRNA methylation in the 3 ‘UTR and nearby stop codons, which selectively methylates primarily by recruiting the catalytic m^6^A core assembly METTL3/METTL14/WTAP guide region (17).

### Erasers

m^6^A demethylases, functioning akin to erasers, eliminate m^6^A modifications in RNA. The initial discovery in 2011 identified the fat mass and obesity-associated protein (FTO) as the pioneer m^6^A demethylase, positioned in both the cytoplasm and nucleus. FTO plays a crucial role in glycolysis and adipogenesis by eradicating m^6^A methylation (18, 19). Another demethylase, alkylated DNA repair protein alkB homologue 5 (ALKBH5), uncovered in 2013, is predominantly localized in the nucleus. ALKBH5 induces conformational changes in Flip 3, expediting nuclear export and influencing mRNA export and metabolism (20, 21). Recent findings indicate that the methyltransferase ALKBH3 predominantly mediates m^6^A modification in tRNA (22).

### Readers

Multiple readers of m^6^A serve as recognition functions during m^6^A methylation and affect the fate of RNA (23). Among them, YT521-B homology domain-containing family (YTHDF) is one of the main players (24). The YTHDF family consists of YTHDF1-3 and YTHDC-2. Within this family, YTHDF1-3 serve distinct roles in m^6^A modification. YTHDF1 interacts with translation initiation factors, enhancing mRNA translation. YTHDF2 hinders the binding of mRNA stability proteins, facilitating the degradation of 3′ UTR m^6^A-modified mRNA. Meanwhile, YTHDF3 both promotes mRNA translation alongside YTHDF1 and mediates mRNA degradation in collaboration with YTHDF2 (25–27). YTHDC2 is an RNA helicase whose structure can aid RNA binding, is involved in mRNA regulation, and is particularly highly expressed in germ cells (28). YTHDC1 is able to interact with transcripts containing m^6^A and regulate downstream targeted gene expression (29).

## Biological function of m^6^A

Certainly, m^6^A assumes a pivotal role in diverse biological functions, participating in various facets of RNA metabolism. This comprehensive involvement influences the entirety of the RNA life cycle, regulating a myriad of cellular processes (30). Among them, m^6^A is involved in multiple aspects of mRNA, including splicing, nuclear export, translation, and RNA stability (14, 31, 32) (**Figure 2**).

YTHDC1 regulates mRNA splicing by directly recruiting and regulating pre-mRNA splicing factors, in addition to the abundant nuclear protein HNRNPs that regulate pre-mRNA processing that can also indirectly affect alternative splicing of mRNA (33). Additionally, the m^6^A-binding protein YTHDC1 has been shown to interact with export proteins serine/arginine-rich splicing factor 3 (SRSF3) and nuclear RNA export factor 1 (NXF1), facilitating the transport of methylated mRNA from the nucleus to the cytoplasm in HeLa cells and playing a role in mRNA nuclear export. In mouse embryonic stem cells, m^6^A-modified mRNAs exhibit reduced stability compared to unmodified transcripts, leading to decreased abundance and translational potential (34).

YTHDF2 has been implicated in influencing the efficiency and stability of RNA translation of m^6^A. Mechanistically, YTHDF2 recruits the CCR4-NOT deadenylase complex, promoting mRNA degradation through a direct interaction between the N-terminal region of YTHDF2 and the SH domain of the CCR4-NOT transcription complex, subunit 1 (CNOT1) subunit. In contrast, in T. brucei, mRNA stability increases when the poly A tail of transcripts is methylated in association with variant surface glycoproteins (35). Moreover, m^6^A plays a crucial role in regulating mRNA translation. On the one hand, m^6^A at the 5’ UTR position enhances translation independently of the cap structure. Mechanistically, m^6^A directly binds to eIF3, initiating translation. On the other hand, at the 3’ UTR, m^6^A interacts with YTHDF1 or YTHDF3, promoting cap-related translation (19, 36, 37).

In rRNAs, novel m^6^A methyltransferases, such as ZCCHC, have been experimentally identified for methylating human 28S rRNA. Knockdown of ZCCHC4 in mammalian cell lines resulted in the elimination of the m^6^A4220 modification in 28S rRNA, leading to reduced global translation in hepatoma cell lines and inhibiting cell proliferation (38, 39).

## Roles of m^6^A modification in immune cell biology

**Figure 3:**
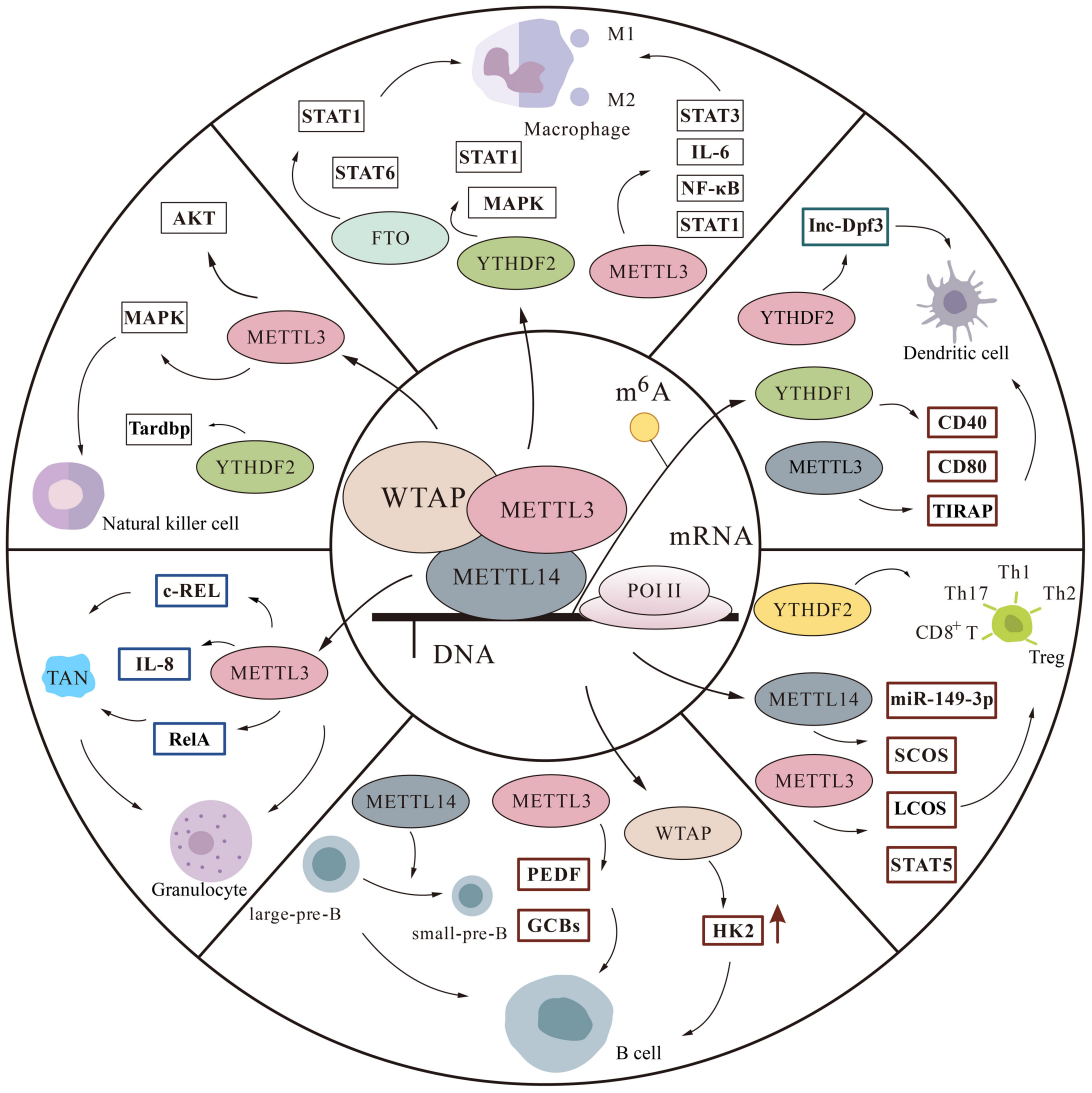
The m^6^A modification is involved in regulatory pathways in immune cells. We mainly describe some protein factor changes of m^6^A in macrophages, monocytes, natural killer cells, T cells, B cells, and granulocytes thereby affecting the immune microenvironment.

The immune system includes innate and adaptive immunity, in which various immune cells perform their duties. Macrophages, natural killer (NK) cells, Dendritic cells (DCs), neutrophils, are involved in immune responses in innate immunity. T cells and B cells are able to specifically clear pathogens and participate in adaptive immunity (40). An increasing number of experiments have demonstrated that m^6^A is involved in regulating the immune microenvironment by mediating various immune cells (**Figure 3**).

### m^6^A and macrophages

Macrophages serve as the frontline of host defense, playing a crucial role in both innate and adaptive immunity. When activated, they not only engulf pathogens but also release cytokines, actively participating in immune responses. Activated macrophages are categorized into M1 and M2. While M1 primarily assumes a pro-inflammatory role, M2 enhances anti-inflammatory factor production, mitigating the inflammatory response (41, 42).

It has been shown that m^6^A is involved in macrophage polarization. Macrophage polarization impairment is a major cause of RA disease progression. Signal transducer and activator of transcription 1 (STAT1) mRNA plays an important role in M1 macrophage polarization. The “writers” protein METTL3 in m^6^A was found to methylate STAT1 mRNA thereby increasing STAT1 levels and promoting M1 polarization, and conversely, knockdown of METTL3 decreased M1 and increased M2 in macrophages (43). METTL3 levels were upregulated in peripheral blood mononuclear cells from RA patients and positively correlated with C-reactive protein (CRP) and erythrocyte sedimentation rate, two markers of RA disease activity. Increased METTL3 may ameliorate inflammation in RA patients by inhibiting nuclear factor kappa-B (NF-κB) signaling to suppress lipopolysaccharide (LPS)-induced expression of inflammatory cytokines Interleukin-6 (IL-6) and tumor necrosis factor alpha-α (TNF-a) in macrophages (44).

Macrophage toll-like receptors (TLRs) have an important role in the immune microenvironment. Once pathogens invade, they trigger a series of intracellular signaling pathways that produce various pro-inflammatory factors and clear pathogens. Upon LPS stimulation *in vitro*, macrophages lacking METTL3 showed loss of m^6^A modification of recombinant interleukin 1 receptor associated kinase 3 (IRAKM) mRNA, decreased TNF-α levels, and ultimately suppressed TLR signaling-mediated macrophage activation (45).

Pulpitis is a bacterial infection in the oral cavity that causes an inflammatory response that can further deteriorate into pulp necrosis and periodontal disease. METTL3-induced m^6^A levels have been found to be elevated in lipopolysaccharide LPS -stimulated human dental pulp cells (HDPCs). Whereas knockdown of METTL3 promotes MyD88S, which is a splice variant of myeloid differential protein-88 (MyD88) and inhibits various inflammatory factors, it alleviates the inflammatory response of HDPCs (46). Furthermore, METTL3 overexpression induces heightened secretion of IL-6 and Inducible Nitric Oxide Synthase (iNOS) in M1 macrophages, while also boosting the osteogenic and migratory capacities of bone marrow stromal cell (BMSC) (47).

Alternatively, METTL14-mediated m^6^A modification is implicated in the regulation of macrophages. Depleting the m^6^A methyltransferase subunit METTL14 in myeloid cells heightened the macrophage response to acute bacterial infection in mice. METTL14 depletion weakened suppressor of cytokine signaling 1 (SOCS1) m^6^A methylation, reducing YTHDF1 binding to m^6^A sites, subsequently diminishing SOCS1 induction and leading to overactivation of the TLR4/NF-κB signaling pathway (48).

In addition, m^6^A can regulate C1q^+^ tumor-associated macrophages (TAM), but also CD8^+^ T cells through some ligands. Knockdown of METTL14 was found to promote CD8^+^ T cell differentiation and impair their function thereby eliminating tumors (49). In a separate study, the knockdown of YTHDF2 significantly increased the expression of inflammatory factors such as IL-6, TNF-α, IL-1β, and IL-12 in LPS-induced macrophages. This resulted in the activation of the mitogen-activated protein kinase (MAPK) and NF-κB signaling pathways, thereby intensifying LPS-induced inflammation in RAW 264.7 cells (50).

These findings imply that m^6^A plays a role in various aspects of macrophage activation and function, suggesting it could serve as a target for improving a range of inflammatory diseases through the regulation of m^6^A modification.

### m^6^A and NK cells

NK cells are critical cells in innate immunity and can non-specifically and directly remove cancer cells and infected cells (51, 52). METTL3 expression was found to be decreased in tumor-infiltrating NK cells, and its protein expression level was positively correlated with NK cell effector molecules. Absence of METTL3 protein imbalances NK cells, thereby inhibiting NK cells from playing a role in immunity, leading to rapid tumor cell growth. This was associated with decreased activity of the m^6^A-modified gene encoding Tyrosine Phosphatase Shp-2 (SHP-2), inhibition of KT-mTOR and MAPK-ERK signaling pathways as well as decreased response to IL-15 and suggests that METTL3-mediated m^6^A promotes NK cells and can alleviate tumor progression, providing new possibilities for cancer immunotherapy in the future (53).

In Testicular germ cell tumors (TGCTs), METTL3 protein levels decreased, tumor growth was evident, and patient survival decreased. The expression of METTL3 was positively correlated with molecular markers and infiltration levels of CD8^+^ and CD4^+^ T cells as well as natural killer cells, as illustrated by this study (54).

Research has highlighted the significance of the “readers” protein YTHDF2 in recognizing and interpreting m^6^A modifications within NK cells, which is critical for maintaining NK cell homeostasis, as well as for their survival and proliferation mediated by IL-15. In response to various stimuli such as tumor cells and infections, the expression of YTHDF2 increases in NK cells. Its absence leads to decreased antitumor and antiviral ability of NK cells. The role of YTHDF2 in NK cell function is associated with its interaction with TAR DNA-binding protein (Tardbp). The specific binding of YTHDF2 to Tardbp likely plays a critical role in the regulation of NK cell responses (55).

### m^6^A and DCs

DCs have the ability to phagocytose and present antigens. They are responsible for initiating adaptive immune responses, particularly the antigen-specific activation of naive T cells, and serve as a bridge between innate and adaptive immunity (56). Recently, many studies have found that m^6^A is involved in regulating DCs (57).

It has been shown that METTL3-modified m^6^A positively regulates maturation and activation of DCs. CD40, CD80, and TIR domain-containing adaptor protein (TIRAP) are important transcripts in DCs. METTL3-mediated m^6^A methylation of these transcripts enhances translation of DCs and stimulates T cell activation, promoting secretion of relevant cytokines by activating the TLR4/NF-κB signaling pathway (58). In another study, reduced release of major histocompatibility complex II (MHCII), costimulatory molecules (CD80, CD86), and cytokines [interferons-γ (IFN-γ), IL-12] was observed, along with a decreased ability to mediate T-cell activation in DCs that were knocked down for METTL3, exhibiting immature properties (59).

Furthermore, CCR7-induced IncRNA lnc-Dpf3 inhibited CCR7-mediated migration of DCs, and YTHDF2 aggravated the inflammatory response by mediating m^6^A modification of lnc-Dpf3 (60). Studies have shown that m^6^A negatively regulates tumor neoantigen-specific immunity through YTHDF1, and YTHDF1 cross-presents tumor antigens in DCs, thereby stimulating T cell activation (61).

Pathogenic autoantibodies in SLE patients bind to antigens and form immune complexes (ICs) deposited in target organs, which can activate TLR on DCs and produce large amounts of type I IFNs, especially IFN-α and IFN receptors to promote a series of autoimmune responses in CD_4_^+^ T lymphocytes, CD_8_^+^ T lymphocytes and B cells, causing abnormal immune regulation. It has been shown that loss of YTHDF1 leads to recruitment of DCs, increased MHCII expression and IL-12 secretion, promotes penetration of CD_4_^+^ and CD_8_^+^ T cells, and increases IFN-γ secretion, playing a role in disease remission (62).

### m^6^A and T lymphocytes

T lymphocytes play a very important role in adaptive immunity. T cells are mainly divided into CD4^+^ T cells and CD8^+^ T cells, and the imbalance of T cells can cause impaired immune function and cause ADs (63, 64) It has been shown that m^6^A plays a role in maintaining homeostasis of T cells.

Naïve CD4^+^ T cells exhibit the ability to differentiate into distinct cell subsets in response to various stimuli. These subsets encompass CD4^+^ helper T cells, specifically Th1, Th2, Th17 cells, CD4^+^ regulatory T cells (Treg cells), and follicular helper T cells (Tfh cells) (65–67).

The deletion of METTL3 in mouse T cells has been observed to have a significant impact on T cell homeostasis and differentiation. In METTL3-deficient naive T cells, there is a notable increase in the expression levels of mRNA and protein of the SOCS family. This upregulation of SOCS proteins inhibits the IL-7-mediated activation of STAT5, leading to impaired T cell homeostatic proliferation and differentiation (68).

The relationship between enterotoxigenic Bacteroides fragilis (ETBF), inflammatory bowel disease (IBD) and rectal cancer (CRC) has been investigated. They found that METTL14-mediated m^6^A regulated Th17 cell differentiation by mediating exosome miR-149-3p of cells in ETBF-treated cells (69). Similarly, METTL14 deficiency leads to increased Th1 and Th17 cytokines and dysfunctional Treg cells, inducing IBD (70).

Th2-dominant response caused by Th1/Th2 imbalance is one of the important causes of allergic asthma. Five candidate m^6^A regulators [fragile X mental retardation 1 (FMR1), KIAA1429 (VIRMA), WTAP, YTHDF2, ZC3H13] were screened in the Gene Expression Omnibus GSE40888 dataset by a random forest model, and asthmatic children were distinguished into two m^6^A patterns (cluster A and cluster B) using consensus clustering to quantify m^6^A patterns. The results showed that patients in group B had higher m^6^A scores than those in group A. In addition, it was found that patients with group A were associated with Th1 dominant immunity, while patients with group B were associated with Th2 dominant immunity. In summary, m^6^A regulators may be involved in the development of childhood asthma, but the specific mechanism of involvement needs to be further experimentally demonstrated (71).

In addition, m^6^A also maintained the inhibitory effect of Treg cells, and METTL3 deletion resulted in increased SOCS mRNA in Treg cells, which targeted the inhibition of IL-2- STAT5 signaling pathway and finally lost the inhibitory function of Treg cells (72). Tfh cells are T cell subsets that assist B cells in humoral immunity to produce antibodies and play an important role in humoral immunity. There is evidence that m^6^A modification has an inhibitory effect on Tfh cells, mainly by reducing ICOS secretion, a key molecule for Tfh development (73).

CD8^+^ T cells, vital for immune defense, play a crucial role by secreting various cytokines to exert their immune effects. The regulation of CD8^+^ T cell function is closely linked to m^6^A regulation. In a mouse tumor model, mice lacking YTHDF1 exhibited heightened antigen-specific CD8^+^ T cell antitumor capacity (61). Moreover, FTO in tumor cells can suppress the activity of CD8^+^ T cells, enabling tumor cells to evade immune surveillance. Knocking down FTO reduces the glycolytic activity of tumor cells, restoring the functional capabilities of CD8^+^ T cells and effectively blocking immune escape mechanisms, thereby inhibiting tumor growth (74). In tumor-associated macrophages, the absence of METTL14 induces aberrant differentiation in CD8^+^ T cells, compromising functionality and inhibiting activation in CD8^+^ T effector cells (49).

It has been found that the expression of ALKBH5 is down-regulated in both PBMCs and T cells of SLE patients, and ALKBH5 mRNA levels are negatively correlated with SLE disease activity index score, erythrocyte sedimentation rate and anti-dsDNA levels, and positively correlated with complement C3 and C4 levels. Mechanistically, functionally, overexpression of ALKBH5 promoted apoptosis and inhibited T cell proliferation (75).

### m^6^A and B lymphocytes

B lymphocytes are one of the main immune cells in the body and play an important role in humoral immunity. Activated mature B lymphocytes can differentiate memory B cells and plasma cells and produce corresponding antibodies to kill pathogens (76).

According to studies, the lack of the m^6^A “writers” METTL14 significantly interferes with B cell development, impairs IL-7-induced proliferation, and affects the transition from large pre-B to small pre-B cells. The reduced binding of YTHDF2 to its target genes due to the absence of METTL14 is pivotal for the progression from the pro-B stage to the large pre-B stage, which is the first time that m^6^A has been found to be associated with B cell development (77).

The deletion of m^6^A using Mb1-Cre severely impairs B cell development. Interestingly, specifically knocking down METTL3 at the pro-B cell stage shows only minor effects on B cell development and function. Furthermore, this targeted reduction of METTL3 does not influence the fibrotropic activity of B cells in the context of liver fibrosis. This suggests that while m^6^A modifications are crucial for B cell development, the specific role of METTL3 knockdown at the pro-B stage might not significantly affect B cell development or function in certain contexts, such as liver fibrosis (78).

Research has demonstrated the crucial role of METTL14-mediated m^6^A modification in the germinative center (GC) B cell response. Elimination of METTL14 in B cells leads to compromised GC B cell proliferation and flawed antibody responses (79). Recent studies have revealed a significant decrease in m^6^A methylation levels in plasma cells taken from patients diagnosed with multiple myeloma. This decrease is associated with the upregulation of FTO, which targets the heat shock factor 1-heat shock proteins (HSF1-HSPs) pathway, promoting the proliferation, migration, and invasion of multiple myeloma cells. These effects occur through YTHDF2-dependent mechanisms. This demonstrates that the role of m^6^A methylation dysregulation in the pathogenesis and progression of multiple myeloma (80).

In addition, m^6^A was demonstrated to be involved in the progression of diffuse large B-cell lymphoma (DLBCL). In DLBCL patients, m^6^A and METTL3 expression levels were elevated. However, high expression of METTL3 can promote cell proliferation. METTL3 can promote cell proliferation and accelerate DLBCL progression by regulating m^6^A methylation and total mRNA levels of pigment epithelium-derived factor (PEDF) (81).

### m^6^A and granulocytes

Granulocytes are white blood cells with special granules in the cytoplasm, which can be mainly divided into three types: neutrophils, eosinophils and basophils (82). In papillary thyroid carcinoma (PTC), METTL3 is able to cooperate with YTHDF2 to inhibit IL-8 secretion, reduce tumor-associated neutrophil (TAN) recruitment, and inhibit tumor growth using c-Rel and RelA as downstream modification targets (83). Currently, our understanding of the effect of m^6^A on granulocytes is still incomplete, and its specific mechanism of action awaits further investigation.

### m^6^A and antiviral immunity

Viral infection is one of the important killers endangering human health. It has been shown that mRNA levels in the virus and host change markedly during viral infection replication (84). In antiviral immunity, m^6^A methylation can regulate host response to viral infection by affecting cytokine levels, regulating immune cell function, and affecting stress response. Host cells recognize associated molecular patterns (PAMPs) through pattern recognition receptors (PRRs), which initiate innate immune responses, which in turn activate signaling pathways, stimulate cytokine release, and further activate adaptive immune responses (85).

It has been found that m^6^A plays an important role in this process. DEAD-box (DDX) helicase is able to initiate antiviral innate immunity. Following viral infection, DDX46, a nuclear DDX member, was able to recruit ALKBH5 to demethylate some m^6^A-modified antiviral transcripts in macrophages from mice, inhibiting signaling and type I interferon production (86).

m^6^A methylation modification can affect immune cell maturation and activation. In monocytes and macrophages, m^6^A of HIV-1 RNA avoids innate immune sensing in bone marrow cells and weakens wolf virus innate immune responses (87). In NK cells, the m^6^A reader YTHDF2 was upregulated at levels following activation of cytomegalovirus infection. YTHDF2 knockdown disrupted the antitumor and antiviral activity of NK cells, with TAR DNA binding protein (TARDBP) being the YTHDF2 binding target in NK cells (55). In DCs, METTL3-mediated methylation of m^6^A is able to promote maturation of DCs. Mechanistically, METTL3 activates DCs function by promoting m^6^A-modified CD40 and CD80 transcripts and translation of TIRAP, a signaling protein in the TLR4/MyD88 pathway that is important for TLR4 signaling and downstream DCs activation.

Besides, loss of METTL3 renders DCs deficient in their ability to promote T cell proliferation (58). In METTL3 knockout mice, T cells exhibit impaired proliferation and differentiation into effector T cells (68). CD4^+^ T cells, susceptible to HIV infection, demonstrate increased HIV-1 protein and RNA expression, along with enhanced viral replication when YTHDF is overexpressed (88).

Viral infections can trigger cellular stress responses, influencing cellular metabolism. m^6^A methylation plays a role in regulating viral infections by modulating the host cell stress response. Flaviviridae RNA virus infection can alter m^6^A modification of some specific cellular transcripts, including RIO kinase 3 (RIOK3) and Cold-inducible RNA-binding protein (CIRBP), by regulating innate immune sensing and endoplasmic reticulum (ER) tress pathways. During viral infection, m^6^A modification of RIOK3 promotes its translation, while reduced m^6^A modification of CIRBP promotes alternative splicing (89). Research has demonstrated that ER stress responses activated by viral infection are involved in m^6^A changes in RIOK3 or CIRBP. These results suggest that viral infection-induced innate immune responses and ER stress contribute to changes in host mRNA methylation modification levels (90).

## Role of m^6^A in immune diseases

**Figure 4:**
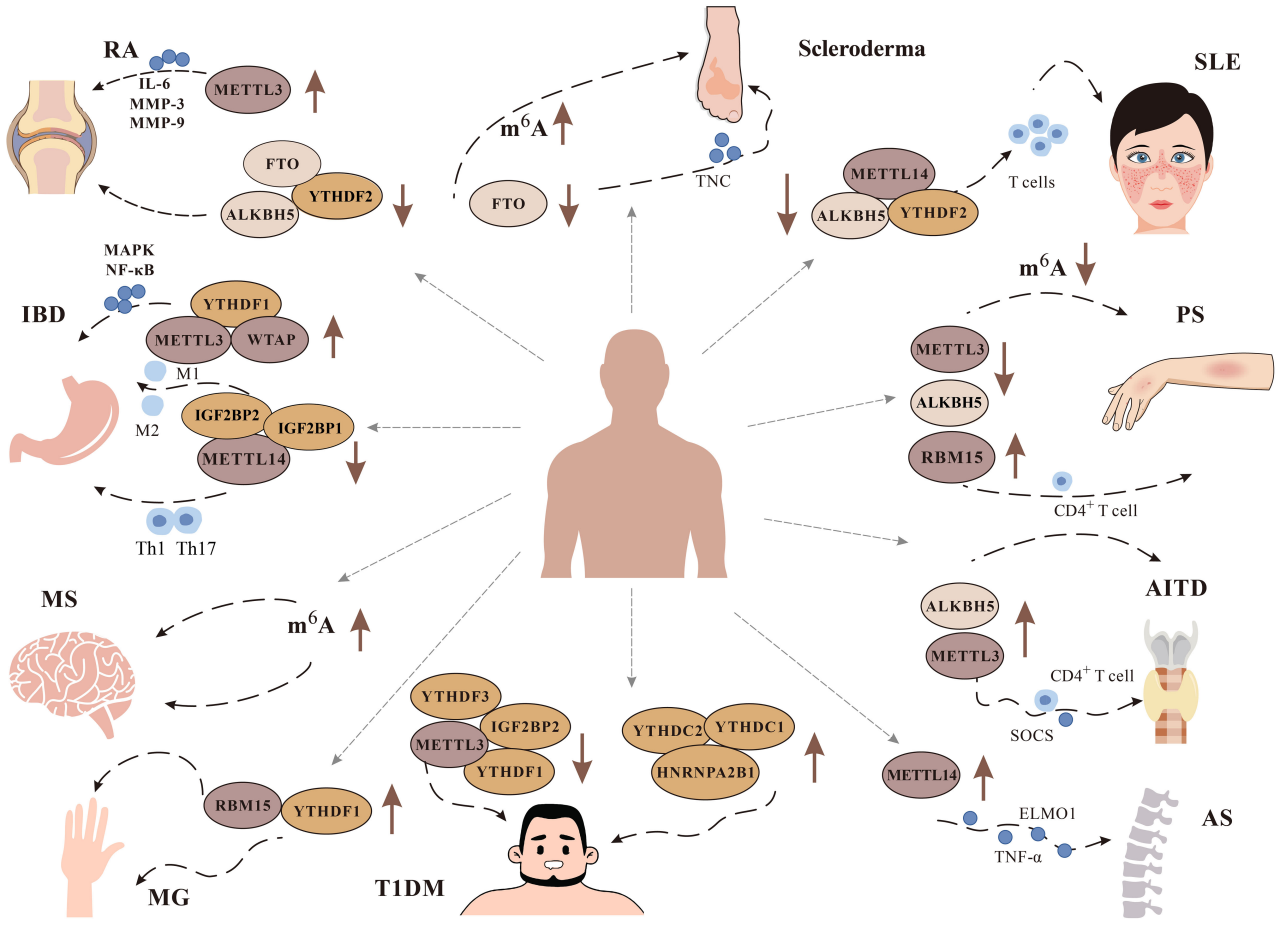
Role of m^6^A modification in immune diseases. It has been found that m^6^A plays a role in a variety of immune diseases, including Rheumatoid arthritis (RA), Systemic lupus erythematosus (SLE), Multiple Sclerosis (MS), Psoriasis (PS), Inflammatory bowel disease (IBD), Type 1 diabetes mellitus (T1DM), Autoimmune thyroid disease (AITD), Ankylosing spondylitis (AS), and Scleroderma. We focus on the changes of related m^6^A methylation-related enzymes METTL3, METTL14, WTAP, YTHDF1, YTHDF2, FTO And ALKBH5 in related diseases.

Excessive inflammation and activation of immune cells can cause tissue damage and organ dysfunction, which leads to ADs (91). The etiology of ADs is not clear, but the role of m^6^A modification in ADs has gradually been discovered (**Figure 4**).

### Rheumatoid arthritis

RA is an autoimmune disease characterized by abnormal proliferation of synovium, which leads to a series of pathological changes such as synovial inflammation and joint injury, and seriously reduces the quality of life of patients (92). Fibroblast-like slippery fetal cells (FLSs) play a crucial role in this (93). It has been found that m^6^A regulates the progression of RA.

SNPs can affect m^6^A by altering RNA sequences or key flanking nucleotides at target sites, and thus m^6^A-associated SNPs (m^6^A-SNPs) may affect RA by regulating genes. A large scale genome-wide association study (GWAS) was performed to screen 37 SNPs associated with m^6^A. Further integration of RA associated m^6^A-SNPs and gene expression data resulted in 23 SNP-Gene-RA trios. However, how m^6^A-SNP impacts RA needs further experimental validation (94). In the clinical cohort, IGFBP2 has been found to be elevated in RA Synovial fibroblasts (SFs). Mechanistically, IGFBP2-regulated neuropeptides growth hormone receptor (GHR) and recombinant natriuretic peptide receptor 2 (NPR2) affect SFs, thereby promoting RA progression (95). In addition, similar studies have demonstrated that IGF2BP3 expression is increased in RA-FLS and regulates G2/M transition to promote RA-FLS proliferation and affect M1 macrophage polarization (96).

Furthermore, the m^6^A regulator ALKBH5 has been shown to be associated with synovial hyperplasia and infiltration. It has been reported that knockdown of ALKBH5 can inhibit the proliferation, migration and invasion of RA FLSs, and mechanistically, ALKBH5-mediated m^6^A can synergistically modify JARID2 mRNA and enhance its mRNA stability with IGF2BP3. The severity of arthritis was also found to be reduced in ALKBH5 knockout delayed-type hypersensitivity arthritis (DTHA) model mice or collagen-induced arthritis (CIA) and rats injected intra-articularly with ALKBH5-shRNA RNA (97). Another study is demonstrating that ALKHB5 may play a role in the activation of RA FLSs regulated by secreted modular calbindin 2 (SMOC2). SMOC2 was significantly increased in RA FLSs and synovial tissue, and knockdown of SMOC2 specifically regulated cytoskeletal remodeling and reduced migration and invasion of RA FLSs. In addition, intra-articular injection of Ad-shRNA-SMOC2 treatment reduced synovial inflammation and reduced erosion of bone and cartilage in CIA rats (98).

In RA patients, expression of METTL3 was significantly increased, as was m^6^A content. Overexpression of METTL3 significantly attenuated LPS-induced macrophage inflammatory responses. Downregulation of METTL3 decreased IL-6, matrix metalloproteinase (MMP) -3, and MMP-9 levels in human RA FLSs and rat AIA FLSs. This process is mediated through modulation of the NF-κB pathway (44, 99).

It has been shown that the content of METTL14 in synovial tissue of RA rats is significantly increased and promotes FLS activation and inflammation in RA rat models. Knockdown of METTL14 significantly increased apoptosis, inhibited cell migration and invasion, and reduced the production of cytokines such as IL-6, IL-18, and CXCL10 in RA FLSs. Mechanistically, METTL14 silencing suppressed LASP1 expression and TNF-α-induced activation of the SRC/AKT axis in FLS, mediating m^6^A modification to improve mRNA stability of LIM and SH3 domain protein 1(LASP1) (100).

Recent studies have found that circ_-_0066715 is less expressed in RA. Circ_-_0066715 regulates the methylation process of WTAP and the expression of the downstream transcriptional gene avian erythroblastosis virus E26 oncogene homolog-1 (ETS-1), affecting macrophage polarization in RA. When circ_-_0066715 was overexpressed, the content of WTAP decreased, increasing ETS1 levels in RA-FLS cells, reducing cytokine secretion on in M1 macrophages, increasing M2 cytokine secretion, and inhibiting FLS proliferation (101).

### SLE

SLE is a systemic inflammatory disease typically caused by immune system abnormalities. Its occurrence is related to genetic factors, environmental factors, estrogen levels and other factors, mainly deposited in the skin, joints, small blood vessels, glomeruli and other parts (102, 103). Recently the role of RNA modification in SLE has gained increasing attention (104).

Decreased mRNA expression of MTEEL14, ALKBH5, and YTHDF2 has been found in peripheral blood mononuclear cells (PBMCs) of SLE patients, and decreased mRNA expression of YTHDF2 has additionally been shown to be a risk factor for SLE according to logistic regression analysis (105).

It has been found by bioinformatics analysis that m^6^A “readers” IGFBP3 and two key immune genes (CD14 and IDO1) of m^6^A may be useful in the diagnosis and treatment of SLE (106). The imbalance of CD4^+^ T cells *in vivo* is one of the important causes of SLE.

In a mouse model immunized with sheep red blood cells (SRBC) and a mouse model of chronic graft-versus-host disease (cGVHD), silencing METTL3 promoted the activation of CD_4_^+^ T cells and the differentiation of effector T cells (mainly Treg cells), increased antibody production and aggravated the lupus-like phenotype in cGVHD mice. Mechanistically, inhibition of METTL3-mediated m6A modification stabilizes Forkhead box protein P3 (Foxp3) mRNA, thereby attenuating Treg differentiation (107).

Type I IFN is the most important cytokine involved in the pathogenesis of SLE, and IFN stimulated gene (ISG) imbalance is an important manifestation of SLE. It has been shown that YTHDF3 inhibits ISG expression by promoting translation of the transcriptional co-repressor forkhead box protein O3 (FOXO3) mRNA, and thus YTHDF3 may play an important role in SLE pathogenesis (108).

At present, there is still a lack of understanding of RNA modification in SLE, but it provides new ideas for the treatment of SLE.

### Multiple sclerosis

MS is a chronic autoimmune disease with inflammatory demyelination of the central nervous system as the main pathological change (109). MS mainly includes progressive multiple sclerosis (PMS) and relapsing-remitting MS (RRMS) (110). Increasing evidence suggests that epigenetic modifications including RNA methylation are involved in MS initiation and progression.

It has been found that 13 central m^6^A RNA methylation modulators are upregulated in MS patients compared with non-MS patients. However, compared with cerebrospinal fluid from RRMS samples and PMS samples, the researchers found that RRMS samples showed significantly higher gene expression levels than m^6^A RNA methylation and m^6^A-related genes. It can be seen that m^6^A methylation is associated with the progression of MS, and can diagnose and distinguish PMS from RRMS (111).

DNA methylation and gene expression are significant risk factors for MS. Experiments integrating data from large-scale genome-wide association studies (GWAS) and quantitative trait loci (QTL) studies through Mendelian randomization have identified mRNA expression for 178 DNA methylation loci and 29 MS-related genes. Among the non-MHC genes identified, METTL21B, METTL1, and the translation elongation factor, mitochondrial (TSFM) showed strong associations. Additionally, MS-associated SNPs in DDR1 were significantly linked to plasma levels of MHC class I peptide-associated sequence B (MICB) and granzyme A. There is a causal relationship between plasma MICB and granzyme A levels and MS. Moreover, rs2288481 in DKKL1 and rs923829 in METTL21B are m^6^A SNPs associated with MS. These SNPs, rs2288481 and rs923829, may influence the regulation of gene expression in DKKL1 and METTL21B, respectively (112).

### Psoriasis

PS is an immune-mediated inflammatory skin disease whose pathogenesis is associated with inflammatory cell infiltration and inflammatory factors. Its etiology is not clear and may be related to genetics, infection, and immune abnormalities (113–115).

RNA methylation has been poorly investigated in psoriasis so far. m^6^A methylation and METTL3 levels show a decrease in psoriatic skin lesions, displaying a negative correlation with psoriasis sarea and severity index (PASI) scores. In experimental models, reducing METTL3 inhibits m^6^A methylation, consequently promoting the development and exacerbating the severity of PS in imiquimod-induced psoriasis-like mice (116). Methyltransferase RNA-binding motif protein 15 (RBM15) has the capacity to modify keratin 17 (K17). Skin samples from PS patients exhibited heightened levels of both RBM15 and K17. When RBM15 was silenced, it hindered proliferation and inflammation by mediating m^6^A modification of K17, thereby reducing K17 stability in IL-17A-induced keratinocytes (117).

m^6^A modification plays a role in PS development through ALKBH5 and METTL3-mediated IL-17a modification in CD4^+^ T cells. In a mouse model of PS, knockdown of ALKBH5 within CD4^+^ T cells aggravated PS-like phenotype and inflammation, and knockdown of METTL3 alleviated this symptom (118).

m^6^A modification is able to play a role in PS by regulating different classes of RNAs. CircRNA is associated with psoriasis. Some research found that a circRNA expressed by macrophages in the inflammatory environment, hsa_-_circ_-_0004287, was upregulated in PBMCs from AD and PS patients. Hsacirc0004287 reduced the stability of its host gene metastasis-associated lung adenocarcinoma transcript 1 (MALAT1) by competitively binding IGF2BP3 to MALAT1 in an m^6^A-dependent manner. MALAT1 levels were low and promoted ubiquitinated degradation of S100A8/S100A9, thereby preventing p38/mitogen activated protein kinase phosphorylation and macrophage-mediated inflammation (119).

lncRNAs have also been found to play a role in PS. Urothelial carcinoma associated 1 (UCA1) was identified as a lncRNA associated with PS and highly expressed in psoriatic lesions. In psoriatic lesions, UCA1 is able to attenuate keratocyte-driven inflammation and PS development by binding to METTL14 and activating hypoxia-inducible factor 1α (HIF-1α) and NF-κB signaling pathways (120). The expression of another lncRNA AGAP2-AS1 is also increased in PS patients and plays a role in promoting the proliferation of keratinocytes and inhibiting apoptosis. m^6^A methylation is involved in its upregulation. METTL3-mediated m^6^A modification suppresses the expression of AGAP2-AS1 via YTHDF2-dependent AGAP2-AS1. In addition, AGAP2-AS1 promotes keratinocyte proliferation by activating the miR-424-5p/AKT/mTOR axis (121). All of the above suggest that m^6^A modification may provide new ideas and directions for the treatment of PS in the future.

### IBD

IBD, including Crohn’s disease (CD) and ulcerative colitis (UC), is a chronic nonspecific intestinal inflammatory disease (122). So far, the etiology of IBD is not clear, but genetic susceptibility, environmental factors and complex interactions between gut microbes play a very important role in it (123).

In recent years, increasing evidence has shown that multiple m^6^A regulators regulate the development of IBD (124). Among them, METTL3 plays different roles in different stages of IBD development. METTL3 overexpression has been found to increase viability, induce apoptosis, increase inflammatory factor release, and aggravate inflammation in epithelial cells treated with LPS (125). Silencing METTL3 reduced the level of TNF receptor-associated factor 6 (Traf6) and inhibited the MAPK and NF-κB signaling pathways, thereby relieving dextran sodium sulfate (DSS)-induced IBD (126).

Dysfunction of T cells is an important cause of IBD. It has been found that METTL14 levels are significantly decreased in IBD patients and promote Th1 and Th17 cytokine secretion, thereby inducing IBD (69).

IGF2BP1 assumes a dual regulatory role in IBD. On the one hand, its absence can foster autophagy in intestinal epithelial cells, nurturing the healing of intestinal inflammation. On the other hand, the absence of IGF2BP1 can diminish the level of occludin, disrupting the delicate balance of the intestinal barrier, hence intensifying the inflammation within the intestine (127, 128).

Furthermore, IGF2BP2 can participate in IBD by mediating macrophage polarization. IGF2BP2-deficient mice have been found to exhibit a more severe inflammatory response in the DSS-induced acute colitis in mice model. This is mainly caused by IGF2BP2 depletion promoting pro-inflammatory M1 macrophage polarization and inhibiting anti-inflammatory M2 macrophage polarization (129, 130).

ALKBH5 acts as an eraser of m^6^A, and its loss can hinder T cell infiltration into colonic tissue and then relieve colitis (131). YTHDF1, as a reader of m^6^A, was significantly increased in intestinal epithelial cells induced by IBD patients and IFN-γ stimulation. Silencing YTHDF1 resulted in decreased pro-inflammatory cytokine release in LPS-stimulated intestinal epithelial cells. Mechanistically, YTHDF1 governs Traf6 mRNA translation. Its loss hinders Traf6 translation, activating NF-κB signaling and the NLRP3 inflammasome, ultimately reducing intestinal inflammation (132, 133).

### Type 1 diabetes mellitus

T1DM is a condition affecting glucose metabolism due to a substantial decrease in insulin secretion, primarily stemming from the extensive destruction of pancreatic β-cells driven by autoimmunity (134). Genetic and environmental factors are the main causes of morbidity (135). In patients with T1DM, there is a notable decrease in the expression of METTL3 and IGF2BP2, while the expression of YTHDC1 and HNRNPA2B1 is significantly increased. Further studies have found that differentially methylated transcripts are involved in some immune pathways closely related to T1DM (136).

Three single nucleotide polymorphisms (SNPs) in non-coding regions of PRRC2A (rs2260051, rs3130623) and YTHDC2 (rs1862315) genes have been found to be closely associated with T1DM. Among them, PRRC2A is increased in T1DM patients, and plays a role in cytokine-cytokine receptor interaction, cell adhesion and chemotaxis, and neurotransmitter regulatory pathways. This process is associated with the PI3K/AKT pathway (137).

Cognitive impairment is a recognized and severe complication of diabetes, particularly in cases of T1DM. Studies in mouse models of T1DM have revealed significant cognitive impairment, with observed alterations in m^6^A-related protein levels within their hippocampus. Specifically, there was notable upregulation in the protein levels of YTHDC2 and ALKBH5, while the expression of YTHDF1, YTHDF3, and WTAP was significantly downregulated. Remarkably, the overexpression of YTHDF1 notably alleviated STZ-induced cognitive dysfunction in diabetic conditions (138).

Islet transplantation is an ideal treatment for T1DM, but hypoxia-induced pancreatic β-cell death after islet transplantation is a huge obstacle leading to this treatment failure. The researchers designed extracellular vesicles (EVs) (HIF-1α-EVs) derived from mesenchymal stem cells (MSCs) with overexpression of HIF-1α. They found that HIF-1α-EV activated autophagy in a YTHDF1-dependent manner, thereby promoting pancreatic β-cells to resist the hypoxic environment and reversing pancreatic β-cell apoptosis and aging (139).

### Autoimmune thyroid disease

AITD, including Graves’ disease (GD) and Hashimoto ‘s thyroiditis (HT), is caused by autoimmune disorders (140). ALKBH5 gene polymorphisms have been found to be associated with AITD, GD, and HT patients, and it is speculated that ALKBH5 may be a candidate gene for AITD susceptibility (141).

Other studies have also found that m^6^A plays a role in AITD. In HT patients, there were significantly more cells in the recombinant activating transcription factor 4 (ATF4) -positive thyroid follicular epithelium (ThyFoEp), which triggered ER stress and led to cell death including cell necrosis and apoptosis. Further studies revealed that HNRNPC as a reader of m^6^A was able to mediate m^6^A modification of ATF4, thereby promoting endothelial cell apoptosis and necrosis (142). Additional experiments have also demonstrated that METTL3 participates in GD by mediating m^6^A modification of SOCS. In GD patients, METTL3 and SOCS molecules are aberrantly expressed in CD4^+^ T cells. Silencing METTL3 increased SOCS family expression (143).

### Ankylosing spondylitis

AS is a chronic inflammatory disease that causes inflammation of the axial joints, leading to tendon ligament calcification and synovial tissue proliferation (144). Evidence suggests that m^6^A modification is reduced in T cells from AS because METTL14 levels are decreased in AS patients, leading to reduced FOXO3a expression, diminished autophagy, and exacerbated inflammation (145).

Recently, it has been found that TNF-α induces enhanced directional migration of AS-mesenchymal stem cells (AS-MSCs) *in vitro* and *in vivo* and promotes AS pathogenesis. In AS-MSC, TNF-α triggers elevated engulfment and cell motility 1(ELMO1) expression, while METTL14 governs MSC migration by influencing specific m^6^A modification sites in ELMO1’s 30 UTR (146).

### Scleroderma

Scleroderma, a connective tissue disease primarily impacting the skin and mucosal tissues, is marked by fibrosis and sclerosis. In a scleroderma mouse model, there was a notable increase in methylation of Hras, Lama3, and Tnc, while methylation of Ccl3, Ccl9, Saa1, and Il1b exhibited a significant decrease. This suggests an association between m^6^A methylation and scleroderma, although the precise underlying mechanism remains elusive (147).

The aggregation of Tenascin C (TNC) is widely recognized as a significant contributor to collagen deposition in the development of scleroderma. Several studies have identified a connection between TNC and m^6^A methylation. In a bleomycin (BLM)-induced scleroderma mouse model, overexpression of FTO led to reduced m^6^A and mRNA levels of TNC, resulting in the alleviation of mouse skin fibrosis. FTO/TNC may become a new target for the treatment of Scleroderma in the future (148).

### Myasthenia gravis

MG is one of the ADs in which lesions occur at the neuromuscular junction (NMJ) of skeletal muscle (149). The m^6^A-related modulators CBLL1, RBM15, and YTHDF1 have been found to be upregulated in MG patients, and primarily target histone-rich modifications and Wnt signaling pathways. Among these, RBM15 showed the strongest association with immune profiles (150).

### Autoimmune hepatitis

AIH is an inflammatory disease of the liver caused by unexplained immune abnormalities, severe cases may develop cirrhosis, liver failure, and even lead to death (151). YTHDF2 expression has been found to be significantly upregulated in AIH patients. Deletion of YTHDF2 in mice led to a progressive rise in bone marrow-derived suppressor cells (MDSC) within the liver. This effect mitigated concanavalin-induced liver injury, enhanced liver expansion, chemotaxis, and ultimately alleviated AIH (152).

### Experimental autoimmune encephalomyelitis

EAE is one of the ADs mainly mediated by specifically primed CD4^+^ T cells, and the cause of the disease is not yet clear (153). Reducing ALKBH5 resulted in heightened m^6^A modification on IFNG and recombinant human C-X-C motif chemokine 2 (CXCL2) mRNA, impairing CD4^+^ T cell responses and contributing to the suppression of autoimmunity (131).

### Celiac disease

CD is a persistent, systemic autoimmune condition affecting the small intestine. It arises from the consumption of gluten-containing cereals by individuals genetically predisposed to the disease (154). The 5 ‘UTR of XPO1 RNA in CD patients has higher YTHDF1 protein-mediated m^6^A methylation and activates downstream NF-κB activity, forming a novel m^6^A-XPO1-NF-κB pathway that causes inflammation (155).

## Potential treatment and application of m^6^A

In recent years, the incidence of ADs has become one of the important causes of damage to human health, and seeking effective therapies for the treatment of ADs has attracted increasing attention. In the above, we focus on the important role-played by m^6^A RNA methylation in the immune microenvironment, which has also been found to be a potential direction and idea for targeted therapy of ADs.

For RA, the m^6^A reader IGF2BP3 promotes RA-FLS by affecting G2/M transition (96). Tripterygium wilfordii Hook F (TwHF) is one of the drugs for the treatment of RA, and triptolide (TP) is its main active component. TP has been found to have a high binding affinity to IGF2BP3 and TP can reduce mRNA expression of IGF2BP3 in PBMC and rheumatoid arthritis fibroblast-like synoviocyte (MH7A) (156, 157). Therefore, IGF2BP3 may be a potential therapeutic target for TP during RA treatment, but this needs further experimental validation. In addition, it has been shown that miR-223p and miR-149-3p regulate osteoblast differentiation of BMSCs by targeting FTO, so FTO may become a new target for the treatment of RA (158).

The m^6^A-related proteins ALKBH5, METTL14, and YTHDF2 are involved in the regulation of SLE. Moreover, m^6^A-related lncRNAs (Xist, PSMB8-AS1, linc02446) are closely related to SLE, as shown by the downregulation of Xist and PSMB8-AS1 expression in SLE patients and the rise of linc02446 expression. Therefore, investigation of m^6^A modification targeting IncRNA could help to innovate the treatment of SLE (159).

Currently, m^6^A inhibitors mostly target FTO and METTL3, whereas eraser of m^6^A ALKBH5 acts on a variety of ADs, and five ALKBH5 gene variants have been found to be detected in AITD patients; deletion of ALKBH5 reduces T cell infiltration into colon tissue and alleviates IBD; ALKBH5 acts on CD4^+^ T cells, and knockdown of ALKBH5 aggravates PS, while inhibition of ALKBH5 may alleviate EAE. Therefore, studies targeting ALKBH5 will provide new therapeutic directions and ideas for the treatment of a variety of ADs (118, 131, 141).

## Conclusion and future perspectives

The coordination among the “writers” (methyltransferase), “erasers” (demethylase), and “readers” (m^6^A-binding protein) intricately governs m^6^A methylation, shaping the destiny of RNA. Beyond influencing transcription, maturation, localization, translation, and degradation of mRNAs, m^6^A methylation extends its impact to various facets of other RNAs, including snRNAs, snoRNAs, miRNAs, and lncRNAs.

Notably, ADs are due to disruption of immune system balance in the body, while m^6^A can influence disease progression by regulating the immune system. In this article, we focus on summarizing the effects of m^6^A on various immune cells, but the role of m^6^A in them remains controversial, especially m^6^A is a dynamically reversible process. In addition, the immune system is an extremely complex mechanism. At present, the regulation of m^6^A on the immune system is mainly concentrated in several major immune cells, but the related inflammatory factors and pathways need to be further studied.

Currently, our understanding of the relationship between m^6^A and ADs is superficial, and the specific regulatory mechanisms and effects on other ADs need to be further experimentally demonstrated. Additionally, m^6^A methylation is a dynamically reversible process, and its precise modification sites in ADs and complex contradictory action relationships need to be clarified. Our research on m^6^A-targeted ADs is still in the basic research stage. The focus of attention in the future will be on accurately providing clinical treatment and developing targeted drugs.

## Author contributions

YC: Writing – original draft. MLi: Writing – original draft. MLu: Writing – original draft. LL: Writing – original draft. HZ: Writing – review & editing. XL: Writing – review & editing.
